# Cerebral arterial air embolism after endobronchial electrocautery: a case report and review of the literature

**DOI:** 10.1186/s12890-021-01580-w

**Published:** 2021-07-12

**Authors:** Yu-Ping He, Yuan-Ling Liu, Xing-Lin Gao, Li-Hua Wang

**Affiliations:** 1 Department of Pulmonary and Critical Care Medicine, Yunfu People’s Hospital, Huanshi Dong Lu No.120, Yuncheng DistrictYunfu, 527300 China; 2 Department of Pulmonary and Critical Care Medicine, Guangdong Provincial People’s Hospital, Guangdong Academy of Medical Sciences, Guangzhou, China; 3 Guangdong Provincial Geriatrics Institute, Guangzhou, China

**Keywords:** Air embolism, Bronchoscopy, Endobronchial electrocautery, Hyperbaric oxygen therapy

## Abstract

**Background:**

Endobronchial electrocautery is a common and safe therapeutic endoscopic treatment for malignant airway obstruction. Cerebral arterial air embolism (CAAE) is a rare but potentially fatal complication of endobronchial electrocautery.

**Case presentation:**

We present the first case of cerebral arterial air embolism after endobronchial electrocautery. A 56-year-old male with a pulmonary tumour in the right upper lobe received repeated endobronchial electrocautery. During the procedure, he experienced unresponsiveness, hypoxemia and bradycardia, and he developed tetraplegia. Brain computed tomography showed several cerebral arterial air emboli with low-density spots in the right frontal lobe. He received hyperbaric oxygen therapy with almost full recovery, except for residual left-sided weakness.

**Conclusions:**

General physicians should realize that CAAE may be a possible complication of endobronchial electrocautery. Several measures, including avoiding positive pressure, lowering ventilatory pressures if possible, avoiding advancing the bronchoscope to occlude the bronchus and using the non-contact technique, should be used to prevent this devastating complication.

## Background

Therapeutic endoscopic treatment may be considered for use in malignant airway obstruction, such as endobronchial electrocautery, argon plasma coagulation (APC), and thermal lasers. Electrocautery is a common and safe bronchoscopy technique. The main complications of electrocautery include bleeding and airway fire. Cerebral arterial air embolism is an extremely rare but potentially fatal complication of bronchoscopy. Azzola et al. [1] reported a frequency of < 0.02% of cerebral air embolism after bronchoscopy in their institution. Although cerebral arterial air embolism has been shown to be a complication of APC and thermal laser treatments [2], it has never been reported in electrocautery. Herein, we described a patient who developed cerebral arterial air embolism (CAAE) after endobronchial electrocautery.

## Case presentation

**Fig. 1:**
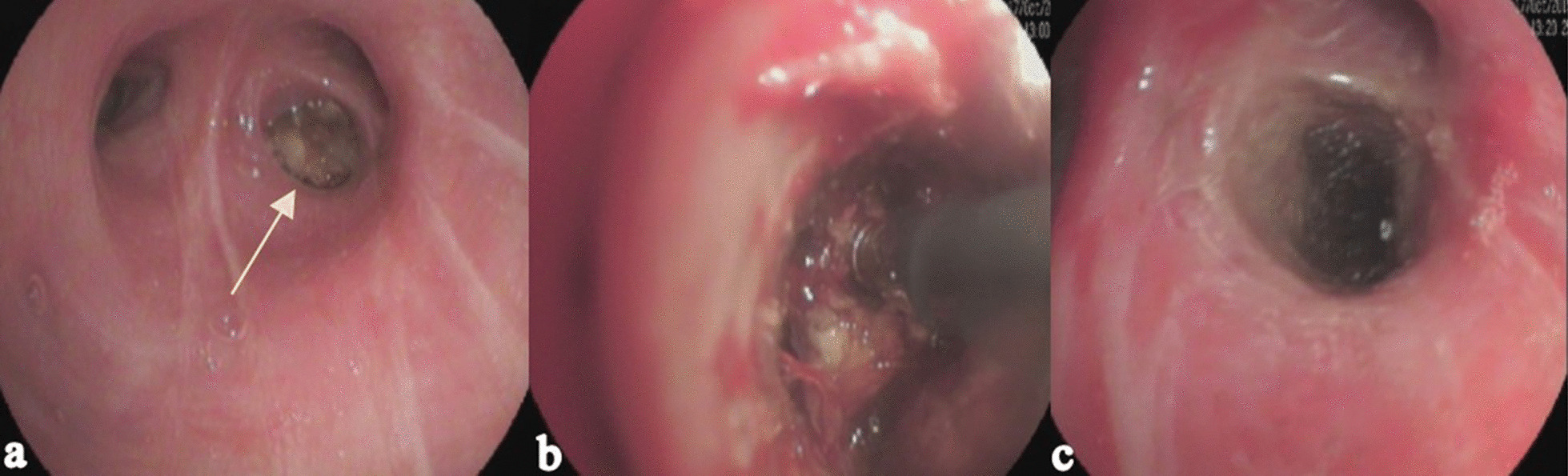
The bronchoscopy showed that the posterior segment of the RUL bronchus was completely obstructed by the tumour (**a**, arrow). Endobronchial electrocautery was used to debulk the tumour (**b**). Bronchoscopy showed the posterior segment after ablation (**c**)

A 56-year-old male, who never smoked, was transferred to our hospital. Approximately 16 years prior, he had been diagnosed with lung cancer and received lung surgery and chemotherapy. Three years ago, he developed a cough and underwent three endobronchial electrocautery treatments in another hospital. As a result, the right middle lobe (RML) mass was debulked. With the diagnosis of the recurrence of right lung endobronchial adenocarcinoma, the patient was admitted for the fourth bronchoscopic thermal ablation procedure to relieve airway obstruction. Bronchoscopy revealed an endobronchial tumour emanating from the posterior segment of the right upper lobe (RUL) bronchus, causing complete obstruction of the bronchus (Fig. 1a). The patient was in the supine position without intravenous sedation. The tumour was debulked using endobronchial electrocautery, and blunt dissection of devitalized tissues with tooth forceps was performed (Fig. 1b, c). The electrocautery cut mode was set at an intensity of 40 W, and the coagulation mode was set at an intensity of 30 W for a duration of 3 s.

**Fig. 2:**
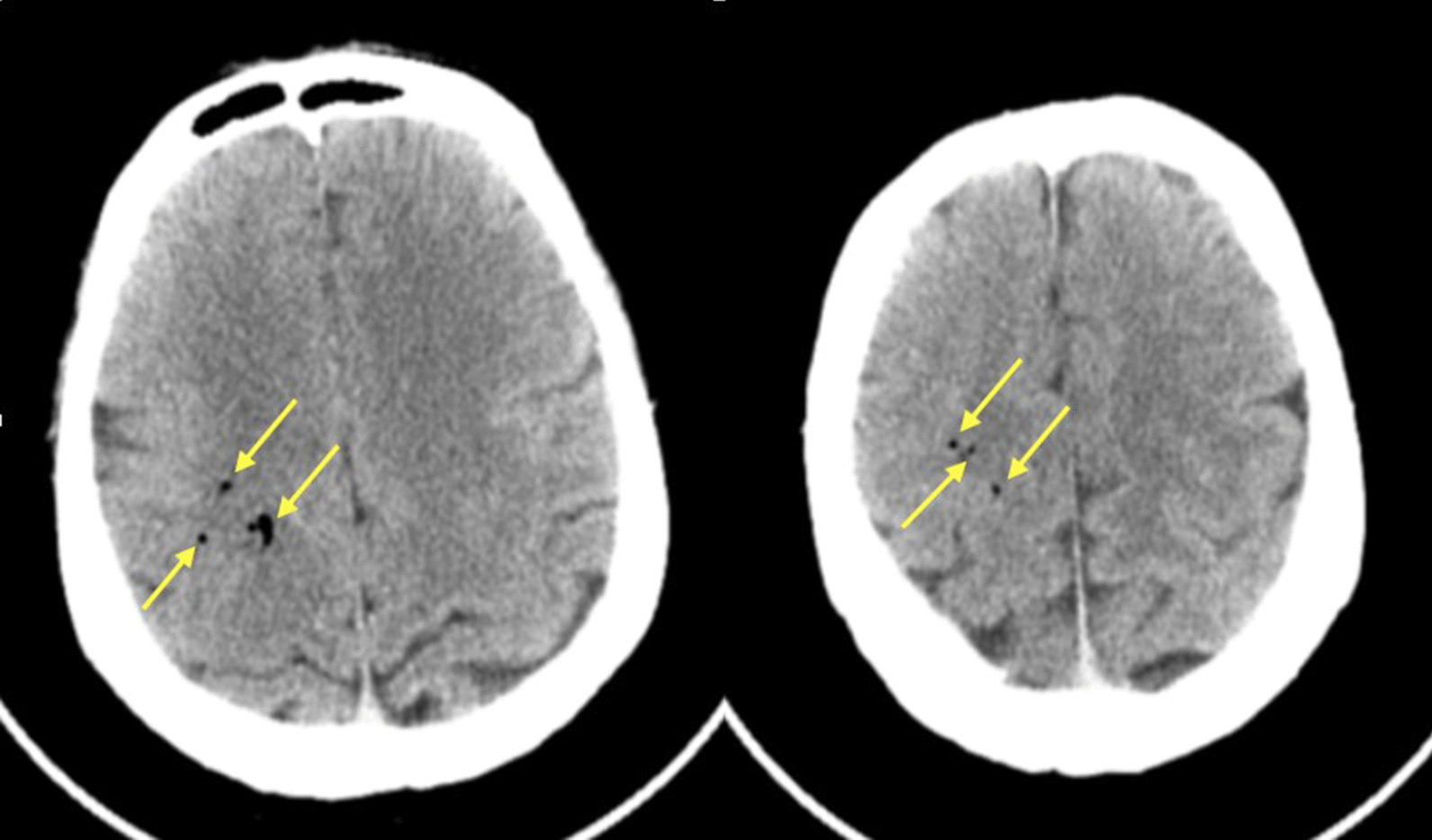
The cerebral CT scan following bronchoscopy showed multifocal cerebral air embolisms in the right frontal lobe (arrows)

The bronchoscopy operation took approximately 30 min in total, including 15 min of endobronchial electrocautery therapy. Fifteen incinerations were performed. During the procedure, the patient showed no cough and no obvious bleeding. At the end of the operation, he suddenly became unresponsive, hypoxemic (SpO_2_ = 88%) and bradycardic (heart rate = 58/min). High-flow oxygen administration with a mask and fluid resuscitation were quickly performed. His heart rate and SpO_2_ recovered quickly, and his consciousness gradually improved 30 min later. However, he presented with obvious abnormal neurological symptoms, including slurred speech, right gaze and paralysis in both extremities. The muscle strength in the left limbs was 0/5, while it was 2/5 in the right limbs. His left Babinski sign was positive. Hypoglycaemia was excluded. Emergency brain computerized tomography (CT) revealed the presence of rounded cerebral gas embolisms that were several millimetres in size and low-density spots in the right frontal lobe (Fig. 2).

**Fig. 3:**
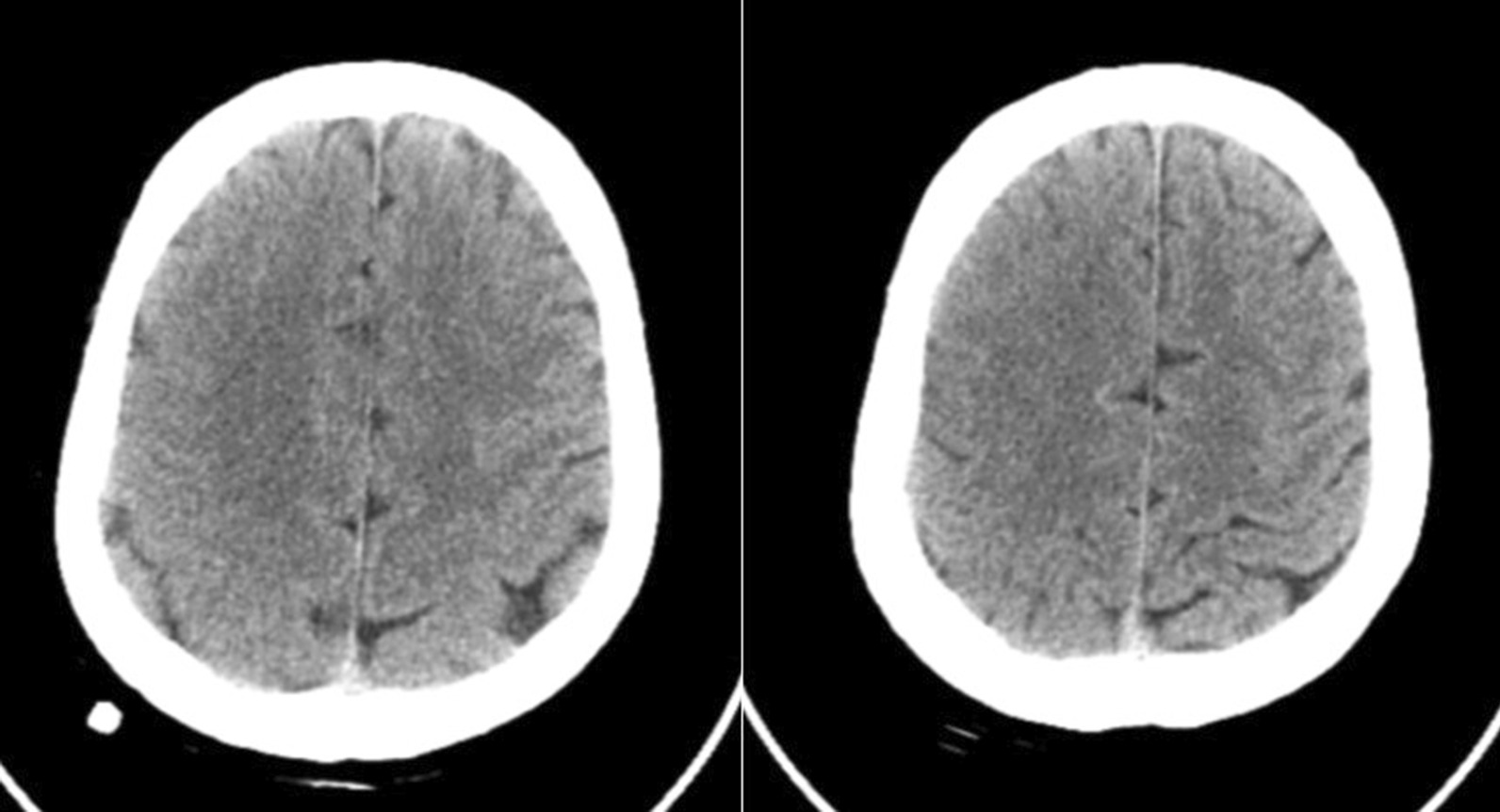
The repeated cerebral CT scan performed 24 h after bronchoscopy demonstrated no signs of air embolism

Hyperbaric oxygen therapy (HBO_2_) was initiated within 2 h after bronchoscopy. He was transferred to the intensive care unit. That night, he received another hyperbaric oxygen therapy. After this treatment, the patient experienced generalized tonic–clonic seizures that were aborted using phenytoin, phenobarbitone and sodium valproate. A repeated brain CT was performed 24 h after bronchoscopy and showed no signs of air embolism (Fig. 3). The patient received intensive rehabilitation. His mental status subsequently improved, but he still had mild left-sided hemiparesis with muscle strength of 3/5 in the left upper limb and muscle strength of 4/5 in the left lower limb.

## Discussion and conclusions

CAAE is a rare but potentially fatal complication of both diagnostic and therapeutic bronchoscopy, and it is often iatrogenic [3]. Because knowledge about the aetiology of CAAE is limited, case reports are therefore important sources to obtain information [4]. To our knowledge, only four cases of CAAE after thermal ablation have been reported [5–8]. The profiles of the reported patients, the examination procedures undertaken, the treatments, and the outcomes are shown in Table 1. This is the first reported case in the literature of CAAE developing after endobronchial electrocautery.

**Table 1:** The profile of patients who developed CAAE after thermal ablation for endobronchial tumour

Reference number	[5]	[6]	[7]	[8]	
Author and year	Osseiran et al	Mimy et al	Yasmeen et al	Kanchustambham et al	This case
	2008	2011	2012	2017	
Patient characteristic					
Age	64	48	88	68	56
Sex	Male	Male	Female	Male	Male
Bronchoscopy					
Procedure	APC	Thermocoagulation	APC	APC	Electrocautery
Location	RML	LUL	RUL	Bronchus intermedius	RUL
Positioning	N/A	N/A	Semi-recumbent	Semi-recumbent	Supine
Bleeding	N/A	> 200 mL	100 mL	Middle	Little
Sedation	Midazolam	N/A	N/A	Fentanyl, Midazolam	(-)
Diagnosis, treatment and outcome of CAAE					
Air bubbles in the CT images	(+)	(+)	(+)	(-)	(+)
Echocardiography shows air	N/A	(–)	N/A	(–)	N/A
Oxygen delivery	Intubation	Intubation, HBO2	Intubation	NBO_2_, HBO_2_	HBO_2_
Seizure	(+)	(+)	(+)	(+)	(+)
Outcome	Dead	Dead	Dead	Almost improved	Almost improved

*APC* argon plasma coagulation, *RML* right middle lobe, *LUL* left upper lobe, *RUL* right upper lobe, *N/A* not available, *HBO*_*2*,_ hyperbaric oxygen, *NBO*_*2*_ normobaric oxygen

The possible mechanisms responsible for the development of CAAE during therapeutic bronchoscopy include the formation of a broncho-vascular fistula, the occlusion of a bronchus by bronchoscopy and paradoxical embolization [3, 4, 8, 9]. A broncho-vascular fistula can form due to inflammation or heat coagulation and mechanical destruction of the tumour and adjacent tissue [7, 10]. In this case, electrocautery ablation of the tumour accompanied by mechanical debridement of the bronchus might have resulted in the formation of the broncho-vascular fistula [1, 11]. Gas can enter the circulation through the fistula, and a gas embolism can easily enter a broncho-vascular fistula if the internal airway pressure goes up with the bronchoscopy process or when the patient is accepting positive pressure ventilation, coughing or taking a deep breath during the procedure [7, 12–15]. Paradoxical embolization occurs when the gas migrates from the venous circulation into the arterial system via an intracardiac shunt (patent foramen ovale)[16]. The air can be eliminated through diffusion into the alveoli, but if the capacity of the air exceeds 50 mL, it easily enters the pulmonary veins [16, 17]. In an animal study, Feller-Kopman et al. [10, 13] found that bronchoscopic thermal ablation with gas flow is associated with the occurrence of gas embolism in a dose-dependent manner, which is consistent with the fact that high flow of coolant gas can lead to vascular air embolism during the use of a contact probe [10]. Other possible risk factors for CAAE include patients with chronic obstructive pulmonary disease, a cavity inside the mass, lesions in the upper lobe, left lateral position, semi-recumbent position and bleeding [1, 7, 18]. In this case, the tumour was located in the posterior segment of the right upper lobe, completely obstructing the bronchus. As the bronchial electrocautery continued, the pressure of the internal airway increased accordingly, which made it easier for air to enter the broncho-vascular fistula (Fig. 1). The increasing pressure of the bronchus during the endobronchial electrocautery procedure may have caused CAAE.

Iatrogenic CAAE can be caused by a broad range of procedures, such as central venous catheter, cardiopulmonary bypass, bronchoscopy, lung biopsy and Nd-YAG laser resection of tumours [4, 17]. In 1979, CAAE was suggested as a complication of transbronchoscopic lung biopsy [12], suggesting that CAAE should be considered a possible cause of neurological symptoms after any procedure with a possibility of CAAE. Clinicians should keep in mind the possibility of CAAE after endobronchial electrocautery.

The symptoms of CAAE develop suddenly and are the same as those of cerebral haemorrhage or thromboembolism, including hemiparesis, tetraplegia, seizures and coma with cardiovascular collapse. In some reported cases, the diagnosis of CAAE should be suspected when there is protracted recovery from sedation [8]. In this case, the patient lost consciousness. The symptoms of CAAE could be fatal or asymptomatic, indicating that it is important to consider the condition even without symptoms [4]. Because asymptomatic CAAE exists, the actual incidence is higher than that previously reported.

Imaging of the brain is useful to visualize air bubbles. In this case, CT was performed within 2 h of symptom onset, and air bubbles were observed in the right frontal lobe. CT may not always reveal the presence of air in the brain as bubbles may be reabsorbed quickly if there is a delay in imaging or if inadaptable window settings are used on non-contrast CT [19]. When the air bubble diameter is less than 1.3 cm, it may not be detected by CT [15]. Kanchustambham et al. [8] reported that air bubbles cannot be visualized on CT images, thereby requiring the diagnosis to be made by exclusion. In this case, the repeated CT after 24 h showed no signs of air bubbles. Hence, CAAE cannot be ruled out through normal imaging. Acute infarction can be confirmed by MRI, which is more time-consuming than CT and might delay therapy. Transoesophageal echocardiography and precordial Doppler are used to detect intravenous and intracardiac air embolisms, and end-tidal carbon dioxide may be an early sign of air embolism [9, 13]. As a result, clinical evaluation is still preferred for the assessment of CAAE [8].

Hyperbaric oxygen therapy is recommended as the most beneficial treatment for CAAEs [9, 16–18, 20]. The mechanisms of hyperbaric oxygen therapy for CAAE include reduced air volume, increased diffusion gradient out of the bubbles, reduction in cerebral oedema, decreased endothelial damage and promotion of restoration of distal blood flow [3, 6, 16, 20]. In some hospitals, HBO_2_ is not available due to the lack of a hyperbaric chamber or the inability to transport patients [1, 7, 15, 18]. The time when the HBO_2_ treatment is started determines the outcome of the patients. Patients treated with HBO_2_ within 5 to 7 h from symptom onset have better outcomes [3, 4, 20]. However, there have been several reports of good outcomes in patients with delayed onset of treatment from 40 h to multiple days [4, 6, 21]. Our patient received the first HBO_2_ treatment within 3 h and the second HBO_2_ treatment within 24 h, and no signs of air bubbles were observed on the repeated CT. The mortality in CAAE patients without HBO_2_ treatment is 93%, while it is 7% with HBO_2_ treatment [21]. In conclusion, HBO_2_ should be initiated as soon as possible after the diagnosis of CAAE to reach the best effect. When there is no available HBO_2_, normobaric oxygen (NBO_2_) should be administered as the most frequent non-HBO_2_ treatment [8, 18].

There are several different opinions on the position of the patient to prevent air embolism. Kanchustambham et al. [9] suggested using left lateral decubitus and the Trendelenburg position. The Trendelenburg position has been reported to reduce air bubbles entering into the brain [20]. However, some studies have revealed that the Trendelenburg position may worsen cerebral oedema, and patients should be placed flat and supine in cases of arterial air embolism [4, 16].

Patients suspected of being diagnosed with air embolism should be transferred to the intensive care unit for careful monitoring and management. Other treatments include endotracheal intubation, volume expansion and extracorporeal membrane oxygenation (ECMO) [9, 22]. Targeted temperature management (TTM) may be helpful to prevent the deterioration of cerebral function in cases of CAAE [23]. Other organs should be examined by echocardiography, electrocardiogram or other examinations when encountering a patient with CAAE because systemic air embolisms should be considered [24].

This is the first reported case of CAAE after endobronchial electrocautery. Physicians should realize that CAAE may be a possible complication of endobronchial electrocautery. Several measures, including avoiding positive pressure, lowering ventilatory pressures if possible, setting the flow to the lowest rate possible when using Nd-YAG or APC, avoiding advancing the bronchoscope to occlude the bronchus and using the non-contact technique, should be used to prevent this devastating complication.

## Abbreviations

CAAE: Cerebral arterial air embolism
APC: Argon plasma coagulation
RML: Right middle lobe
LUL: Left upper lobe
RUL: Right upper lobe
N/A: Not available
HBO_2_: Hyperbaric oxygen
NBO_2_: Normobaric oxygen
CT: Computerized tomography
HBO_2_: Hyperbaric oxygen therapy
ECMO: Extracorporeal membrane oxygenation
TTM: Targeted temperature management
Nd-YAG: Neodymium-doped Yttrium Aluminium Garnet
